# Regulated Expression of an Essential Allosteric Activator of Polyamine Biosynthesis in African Trypanosomes

**DOI:** 10.1371/journal.ppat.1000183

**Published:** 2008-10-24

**Authors:** Erin K. Willert, Margaret A. Phillips

**Affiliations:** Department of Pharmacology, University of Texas Southwestern Medical Center at Dallas, Dallas, Texas, United States of America; Washington University School of Medicine, United States of America

## Abstract

*Trypanosoma brucei* is the causative agent of African sleeping sickness. The polyamine biosynthetic pathway has the distinction of being the target of the only clinically proven anti-trypanosomal drug with a known mechanism of action. Polyamines are essential for cell growth, and their metabolism is extensively regulated. However, trypanosomatids appear to lack the regulatory control mechanisms described in other eukaryotic cells. In *T. brucei,* S-adenosylmethionine decarboxylase (AdoMetDC) and ornithine decarboxylase (ODC) are required for the synthesis of polyamines and also for the unique redox-cofactor trypanothione. Further, trypanosomatid AdoMetDC is activated by heterodimer formation with a catalytically dead homolog termed prozyme, found only in these species. To study polyamine regulation in *T. brucei*, we generated inducible AdoMetDC RNAi and prozyme conditional knockouts in the mammalian blood form stage. Depletion of either protein led to a reduction in spermidine and trypanothione and to parasite death, demonstrating that prozyme activation of AdoMetDC is essential. Under typical growth conditions, prozyme concentration is limiting in comparison to AdoMetDC. However, both prozyme and ODC protein levels were significantly increased relative to stable transcript levels by knockdown of AdoMetDC or its chemical inhibition. Changes in protein stability do not appear to account for the increased steady-state protein levels, as both enzymes are stable in the presence of cycloheximide. These observations suggest that prozyme and ODC are translationally regulated in response to perturbations in the pathway. In conclusion, we describe the first evidence for regulation of polyamine biosynthesis in *T. brucei* and we demonstrate that the unique regulatory subunit of AdoMetDC is a key component of this regulation. The data support ODC and AdoMetDC as the key control points in the pathway and the likely rate-limiting steps in polyamine biosynthesis.

## Author Summary

Human African trypanosomiasis (HAT) is an important vector-borne pathogen. The World Health Organization estimates that more than 50 million people are at risk for the disease, which occurs focally, in remote regions, and periodically reaches epidemic levels. Untreated HAT is always fatal, and the available drugs compromise toxicity and emerging resistance. The only safe treatment for late-stage disease is an inhibitor of an essential metabolic pathway that is involved in the synthesis of small organic cations termed polyamines. In this paper, we use genetic approaches to demonstrate how the parasite regulates this essential metabolic pathway. By modulating the protein levels of a trypanosome-specific activator of polyamine biosynthesis, the parasite has developed a mechanism to regulate pathway output. We also demonstrate that this pathway activator is essential to parasite growth. Our data strengthen the genetic and chemical validation of a key enzyme in this pathway as a drug target in the parasite, and they provide new insight into parasite-specific approaches that could be used to design novel drugs against this deadly disease.

## Introduction

Human African trypanosomiasis is a neglected disease of sub-Saharan Africa caused by the protozoan parasite *Trypanosoma brucei*. Current estimates are that more than 50 million people are at risk for infection [1]. Without treatment the disease is always fatal and available drug therapy is limited by toxicity, difficult dosing regimes, and emerging resistance [2]. Eflornithine (D,L-α-difluoromethylornithine), a suicide inhibitor of ODC, is one of only two drugs available for the treatment of the late-stage disease, and its effectiveness has focused attention on the importance of the polyamine biosynthetic pathway for parasite growth.

**Figure 1:**
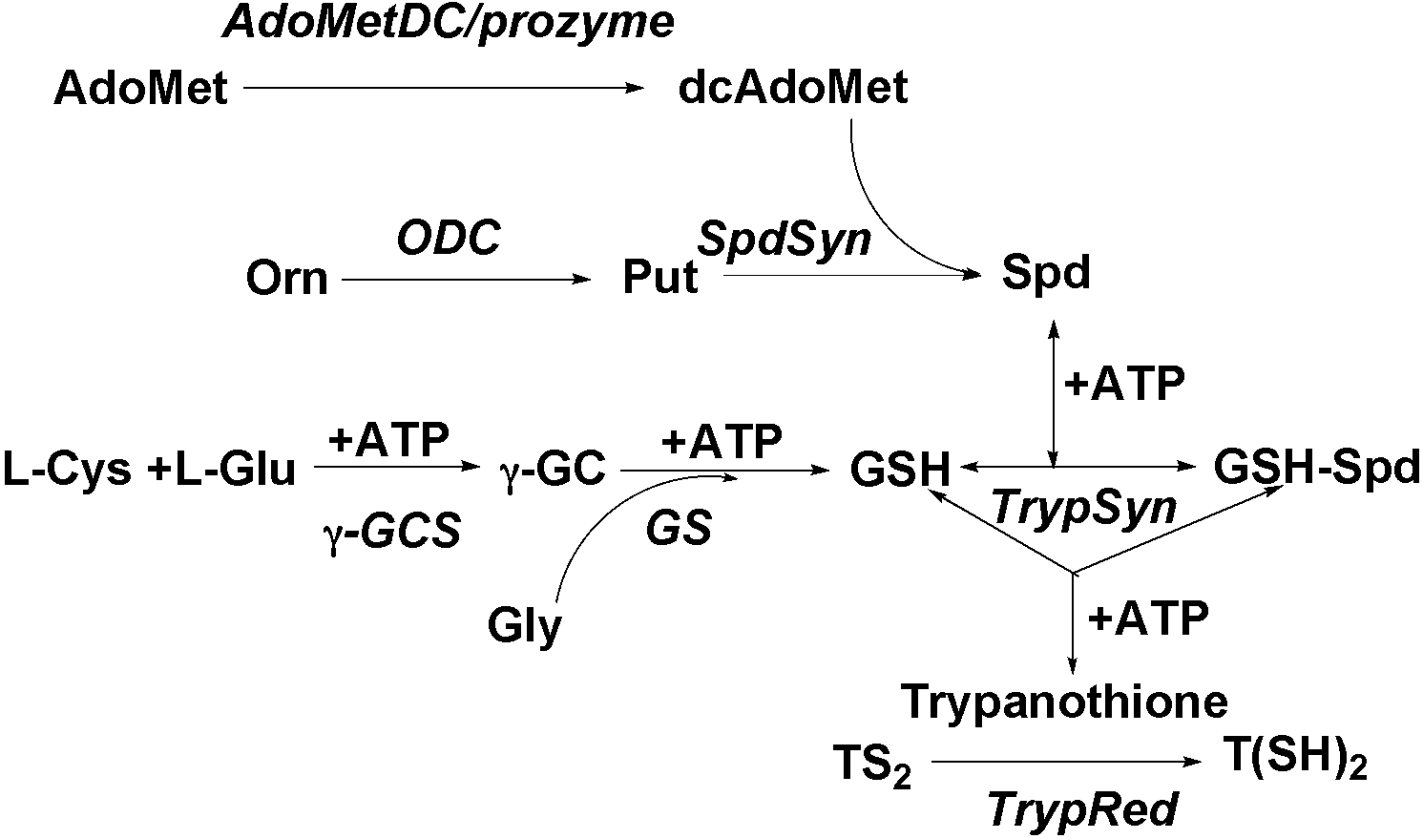
The polyamine biosynthetic pathway in *T. brucei*. AdoMetDC, S-adenosylmethionine decarboxylase; ODC, ornithine decarboxylase; SpdSyn, spermidine synthase; γGCS, γ-glutamylcysteine synthetase; TrypSyn, trypanothione synthetase; TrypRed, trypanothione reductase; Spd, spermidine; Put, putrescine; AdoMet, S-adenosylmethionine; γGC, γ-glutamylcysteine; GSH, glutathione; GSH-Spd, glutathionyl spermidine; TS_2_, oxidized trypanothione; T(SH)_2_, reduced trypanothione.

Polyamines are essential organic cations found in all species, and the metabolic pathway has been extensively studied as a potential target for the development of drugs to treat infectious and proliferative diseases [3],[4]. In most eukaryotes the diamine putrescine is synthesized from L-ornithine by ODC, and it serves as the precursor for the formation of the longer chain amine spermidine (Figure 1). AdoMetDC catalyzes the formation of decarboxylated S-adenosylmethionine required as the aminopropyl group donor in the formation of the longer chain polyamines. Unique to the trypanosomatids, spermidine is conjugated to glutathione (GSH) to produce trypanothione, which is required in cellular redox reactions and necessary for nucleotide synthesis [5]. Gene knockout studies in both *T. brucei* and *Leishmania* have been reported for several of the polyamine and trypanothione biosynthetic enzymes demonstrating that they are essential for growth [6]–[13]. Genetic studies have not been reported for *T. brucei* AdoMetDC, however several promising *in vivo* trials have shown that AdoMetDC inhibitors cure *T. brucei* infections in mice, providing chemical evidence that AdoMetDC is an important drug target against this pathogen [14],[15].

Polyamine homeostasis is essential for normal cellular function. An excess of polyamines leads to hyperproliferation or tumorigenesis, while polyamine deficiency is associated with cell growth arrest leading to death [3],[4]. As a consequence, polyamine pools in eukaryotic cells are tightly regulated, and cells carefully orchestrate a balance of polyamine biosynthesis, degradation and transport into and out of the cell. In mammalian cells, ODC and AdoMetDC are controlled by transcriptional, translational and post-translational mechanisms [3],[16],[17]. Polyamine levels are also regulated by back converting enzymes and polyamine transport. ODC and AdoMetDC both have rapid intracellular turnover rates in mammalian cells. Polyamines accelerate ODC degradation above this basal rate through the action of a protein inhibitor, antizyme [18]. *T. brucei* does not appear to encode the genes for antizyme nor for the back conversion of polyamines, and it lacks the general transcriptional control mechanisms found in other eukaryotes [19], leaving open the question of how polyamines are regulated in the parasite. Recently, we discovered that *T. brucei* AdoMetDC is activated 1,200-fold (on k_cat_) by dimerization with a catalytically dead paralog we termed prozyme [20]. This mechanism for controlling AdoMetDC activity is unique to the trypanosomatid parasites, and the finding raised the possibility that regulation of prozyme expression could provide a parasite-specific mechanism to control polyamine homeostasis in trypanosomatids.

In order to study the potential for AdoMetDC or prozyme to function as regulators in polyamine biosynthesis we utilized RNA interference (RNAi) or regulated knockout approaches in blood form *T. brucei* parasites to deplete the cells of these proteins. Loss of AdoMetDC or prozyme leads to decreases in spermidine and trypanothione and to cell death. A large compensatory induction in the expression levels of prozyme and ODC was observed after either genetic depletion or chemical inhibition of AdoMetDC. Our data support a translational control mechanism for the regulation of these proteins and they provide the first demonstration that polyamine biosynthesis is regulated in *T. brucei*. Thus AdoMetDC and prozyme appear to play a central role in controlling polyamine homeostasis in the parasite through a mechanism that is not found in other eukaryotic cells. These data suggest that prozyme arose as a mechanism to regulate the polyamine metabolic flux in trypanosomes and they illustrate the multiplicity of regulatory control mechanisms that have evolved in this essential metabolic pathway.

## Results

### AdoMetDC is an essential enzyme in *T. brucei*

**Figure 2:**
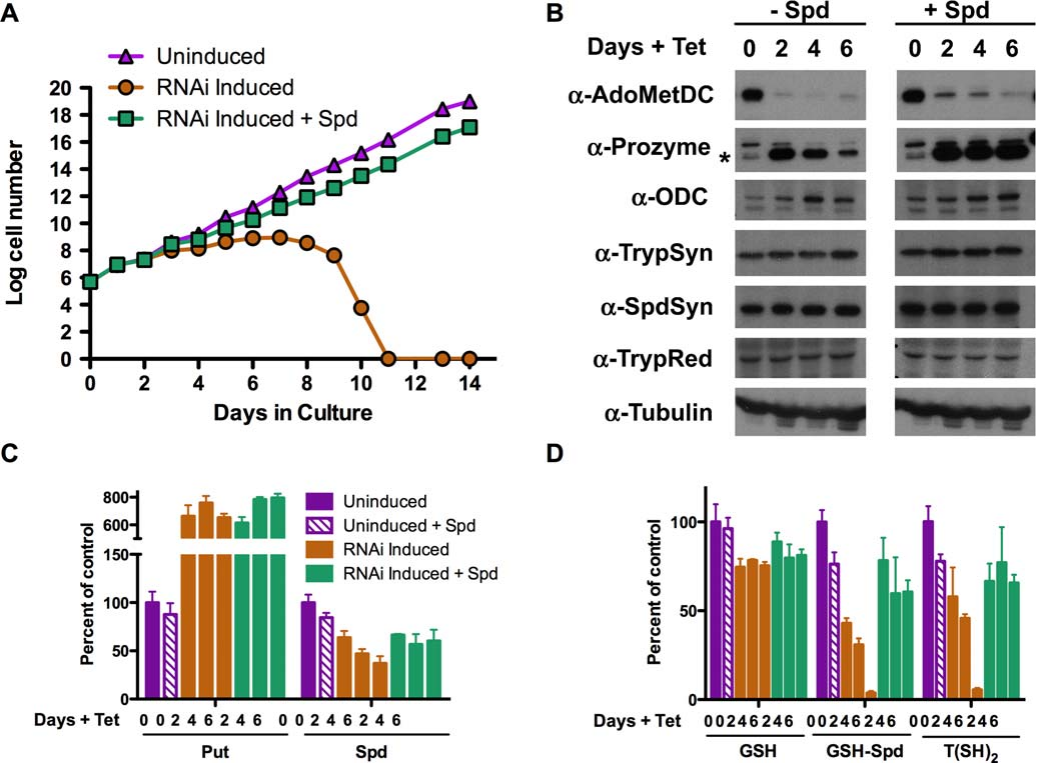
The effects of AdoMetDC knockdown by RNAi on blood form *T. brucei*. (A) Cell growth curves. Log cell number versus days in culture: triangle, uninduced control cells (AdoMetDC RNAi line without Tet); circle, induced cells (AdoMetDC RNAi line +1 µg/ml Tet); square, induced cells plus spermidine (AdoMet RNAi line +1 µg/ml Tet, +0.1 mM Spd). Complete cell death was observed by day 11. Data is displayed in Log units (where log (n) is represented by (n) on the y-axis). (B) Western blot analysis of polyamine/trypanothione biosynthetic enzymes (20 µg total protein). Time corresponds to days post Tet induction of AdoMetDC RNAi, in the absence (left) and presence (right) of spermidine (0.1 mM). The control lane (day 0) on the right panel was cultured in the presence of spermidine for six days prior to the addition of Tet. Prozyme is denoted by (*). (C) Analysis of intracellular polyamine levels. (D) Analysis of intracellular thiol levels. The polyamines Put and Spd, and thiols, GSH, GSH-Spd, and T(SH)_2_ are shown as a percentage of control values (Uninduced AdoMetDC RNAi line). The concentration data (nmoles/10^8^ cells) for all time points and conditions are provided in Table S1. Errors represent the standard error of the mean (n = 3).

To test the effects of reduced AdoMetDC expression on blood form *T. brucei* parasites, we generated a stable cell line with an inducible AdoMetDC targeted RNAi. This line contains a tetracycline (Tet) inducible stem-loop vector with 620 bp fragments of AdoMetDC in opposite orientations integrated into the rRNA gene locus (Figure S1). Addition of Tet leads to production of a double stranded stem-loop RNA targeting AdoMetDC mRNA for degradation. Uninduced AdoMetDC RNAi cells grew at the same rate as the parent 90-13 cells (data not shown). Induction of the AdoMetDC RNAi (+Tet) leads to a reduction in AdoMetDC protein that was maintained until the cells die (Figure 2A and 2B). Cell growth arrest was observed within 4 days of induction, followed by cell death (day 11). Exogenous spermidine (0.1 mM) restored normal growth to the induced cells, demonstrating that the AdoMetDC RNAi specifically targeted spermidine biosynthesis (Figure 2A).

### AdoMetDC depletion reduces polyamine and trypanothione pools

The polyamine and trypanothione metabolite profile was assessed in the AdoMetDC RNAi cells. Intracellular putrescine levels increased 6–7-fold within 2 days of RNAi induction, while spermidine, glutathionyl-spermidine (GSH-Spd) and trypanothione levels gradually declined, with a near complete depletion of GSH-Spd and trypanothione being observed by day 6 after induction (Figure 2C and 2D, and Table S1). The loss of these metabolites correlated well with the point of cell growth arrest. Spermidine was decreased to just below 40% of uninduced controls. Addition of exogenous spermidine to the medium restored the GSH-Spd and trypanothione pools to 70–80% of wild-type levels, while spermidine levels were returned to 60–70% of controls. Under these conditions the cells grow normally. Cell growth could be rescued by spermidine up to four days post-induction, however by day six it was no longer effective (Figure S2). This correlates to the time frame when complete depletion of GSH-Spd/trypanothione is observed (Figure 2D) suggesting that the cells undergo an irreversible event in the absence of reduced GSH-Spd/trypanothione that commits them to death.

### AdoMetDC depletion leads to the induction of prozyme and ODC expression

To determine if AdoMetDC knockdown is coupled to changes in the expression of other enzymes in the pathway, Western analysis of prozyme, ODC, spermidine synthase (SpdSyn), trypanothione synthase (TrypSyn) and trypanothione reductase (TrypRed) was undertaken over the time course of the AdoMetDC RNAi induction (Figure 2B). Prozyme protein levels were markedly increased relative to the tubulin control by day 2 after RNAi induction (Figure 2B). ODC was also consistently higher upon depletion of AdoMetDC. This induction of the prozyme and ODC proteins was observed in the presence and absence of exogenous spermidine, demonstrating that the increased expression is not a result of cell growth effects. No significant effects on the expression of the other pathway enzymes were observed. Control experiments on 90-13 cells (±Tet) demonstrated that Tet had no affect on the expression patterns of any of the tested enzymes (Figure S3).

### Prozyme is essential for growth of *T. brucei*

**Figure 3:**
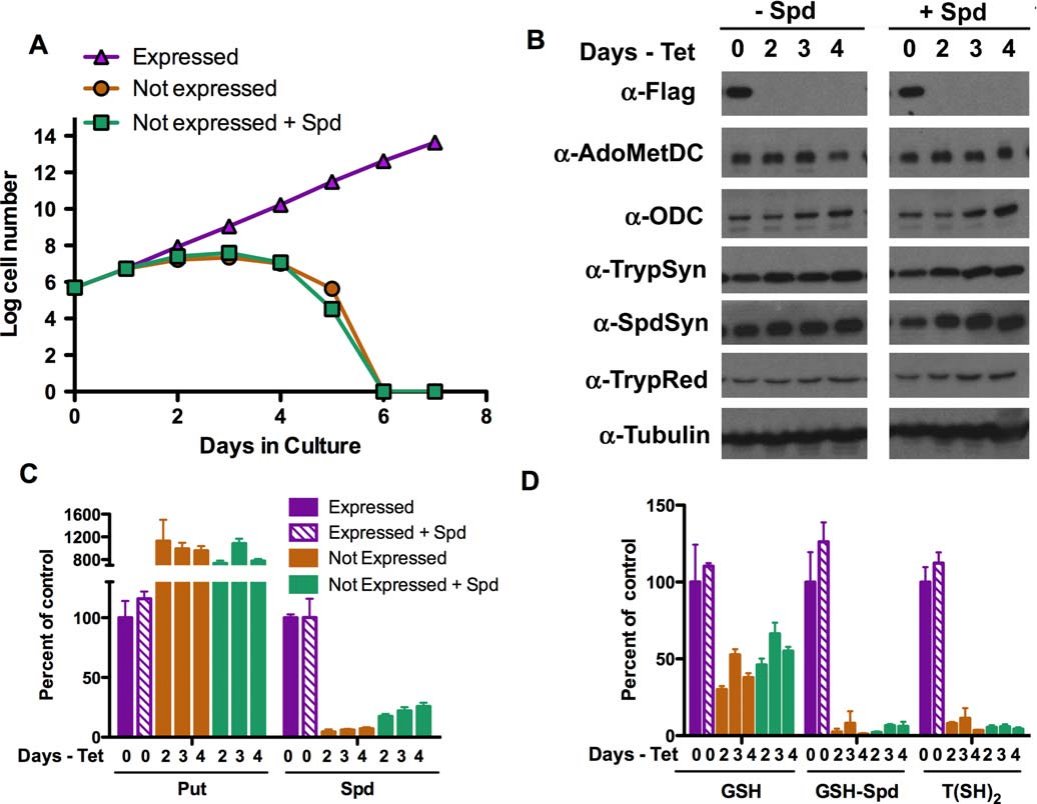
The effects of Prozyme knockout on blood form *T. brucei*. (A) Cell growth curves. Log cell number versus days in culture. Triangle, Flag-tagged prozyme Expressed (prozyme cKO line +1 µg/ml Tet); circle, prozyme Not expressed (prozyme cKO line no Tet); square, prozyme Not expressed plus spermidine (prozyme cKO line no Tet, +0.1 mM Spd). Complete cell death was observed by day 6. Data is displayed in Log units. (B) Western blot analysis of polyamine/trypanothione biosynthetic enzymes (20 µg total protein). Time corresponds to days in the absence of Tet, with (left) or without (right) spermidine (0.1 mM). Flag-antibody detects Flag-tagged prozyme. (C) Analysis of intracellular polyamine levels. (D) Analysis of intracellular thiol levels. The polyamines Put and Spd, and thiols, GSH, GSH-Spd, and T(SH)_2_ are shown as a percentage of control values (prozyme cKO +Tet). The concentration data (nmoles/10^8^ cells) are provided in Table S2. Errors represent the standard error of the mean (n = 3).

The importance of prozyme to growth of blood form *T. brucei* was evaluated by the generation of a prozyme conditional knock out (cKO) cell line. *T. brucei* is a diploid organism, thus to generate the KO line the first prozyme allele was replaced with T7 polymerase and a G418 selectable marker, a Tet responsive FLAG-tagged prozyme gene was integrated into the rRNA locus, and finally the second prozyme allele was replaced by the Tet repressor gene and a hygromycin selectable marker (Figure S1B). Southern blotting confirmed the correct integration of the three vectors (Figure S4). Prozyme cKO cells maintained in the presence of Tet expressed the FLAG-tagged prozyme protein and had similar growth rates to wild-type 427 cells (data not shown). Upon removal of Tet, prozyme expression was reduced to undetectable levels, leading to a rapid arrest of cell growth (day 2) followed by cell death (day 6) (Figure 3A and 3B). Unlike the AdoMetDC RNAi line, the addition of exogenous spermidine did not restore cell growth. These data demonstrate that prozyme is essential for the growth of blood form parasites, and they show that the low activity of homodimeric AdoMetDC (<0.1% of AdoMetDC/prozyme heterodimer activity) is insufficient to promote cell growth. In addition the data confirm that the AdoMetDC/prozyme heterodimer is the functional configuration of AdoMetDC in the cell.

### Prozyme knockout leads to depletion of spermidine and trypanothione

Loss of prozyme in cKO cells resulted in a large increase in putrescine levels (about 10 fold increase), similar to what is seen during AdoMetDC knockdown (Figure 3C and Table S2). However, in these cells a more substantial reduction in spermidine was observed and it was decreased to 5–7% of the levels detected in the uninduced control cells. Similarly to the AdoMetDC RNAi line, GSH-Spd and trypanothione declined to 5–7% of control levels, while GSH pools were 30–50% of controls (Figure 3D). The decline in the GSH pools suggests that part of this pool has been oxidized as the cell attempts to compensate for the loss of reduced trypanothione. Exogenous spermidine partially restored the intracellular spermidine pools (up to 26% of control cell levels); however, the GSH-Spd and trypanothione levels remained below 10%, explaining why the cell growth defect was not rescued.

### Prozyme depletion leads to increased expression of ODC

To look for potential regulation of the polyamine biosynthetic enzymes, we monitored the protein levels of other pathway enzymes by Western blotting in prozyme cKO cells (Figure 3B). Changes in the protein levels of AdoMetDC, TrypSyn, SpdSyn, or TrypRed were not observed in response to the shut down of prozyme expression. However, ODC protein levels increased similarly to that observed upon knockdown of AdoMetDC expression.

### Prozyme is present in limiting concentration under normal growth conditions

**Figure 4:**
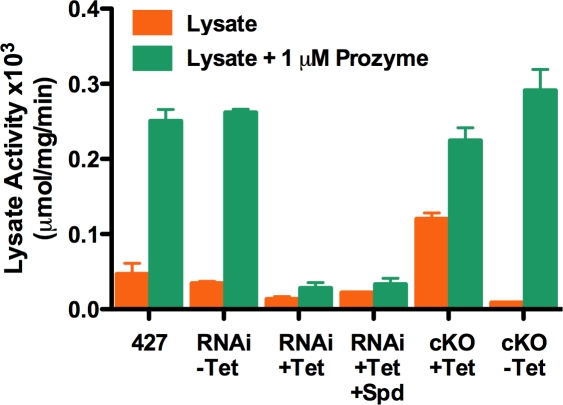
Measurement of AdoMetDC in cell lysates in the presence of excess exogenous prozyme. AdoMetDC activity was measured in the absence (orange) and presence (green) of added recombinant prozyme (1 µM) for 427 parental cells, AdoMetDC RNAi cells (−Tet control and +Tet induction of RNAi for 4 days), and prozyme cKO cells (expressing prozyme (+Tet), and not expressing prozyme (−Tet) collected day 2 after Tet removal). Errors represent the standard error of the mean (n = 3).

In order to determine if AdoMetDC and prozyme are present at similar concentrations, or if one is in excess over the other we measured AdoMetDC activity in cell lysates in the presence and absence of exogenous recombinant prozyme (1 µM). AdoMetDC activity in 427 parental cells was compared to the AdoMetDC RNAi cells (±Tet) and to the prozyme cKO cells (±Tet) (Figure 4). In the control cells (427 cells or AdoMetDC RNAi (−Tet)) the addition of prozyme stimulates AdoMetDC by 5–8-fold, demonstrating that under normal growth conditions prozyme is present in limiting concentration in comparison to AdoMetDC. Knockdown of AdoMetDC (RNAi +Tet) or of prozyme (cKO–Tet), reduced AdoMetDC activity by 60% and 80%, respectively, showing that while neither knockdown approach completely eliminates the protein targets, the prozyme cKO is more efficient at depleting AdoMetDC activity than the induction of AdoMetDC RNAi. The addition of exogenous prozyme has a minimal effect on the activity of the Tet induced AdoMetDC RNAi cells, however activity is increased significantly for the prozyme cKO cells (−Tet), showing that added prozyme can restore activity to the lysate that lacks endogenous prozyme. These data demonstrate that prozyme is present in limiting concentration under typical growth conditions of the wild-type 427 cells, positioning the cell to increase pathway flux by upregulating prozyme levels.

### Quantitative analysis of Prozyme and ODC protein and mRNA levels: Expression levels of protein but not mRNA are upregulated by AdoMetDC knockdown

**Figure 5:**
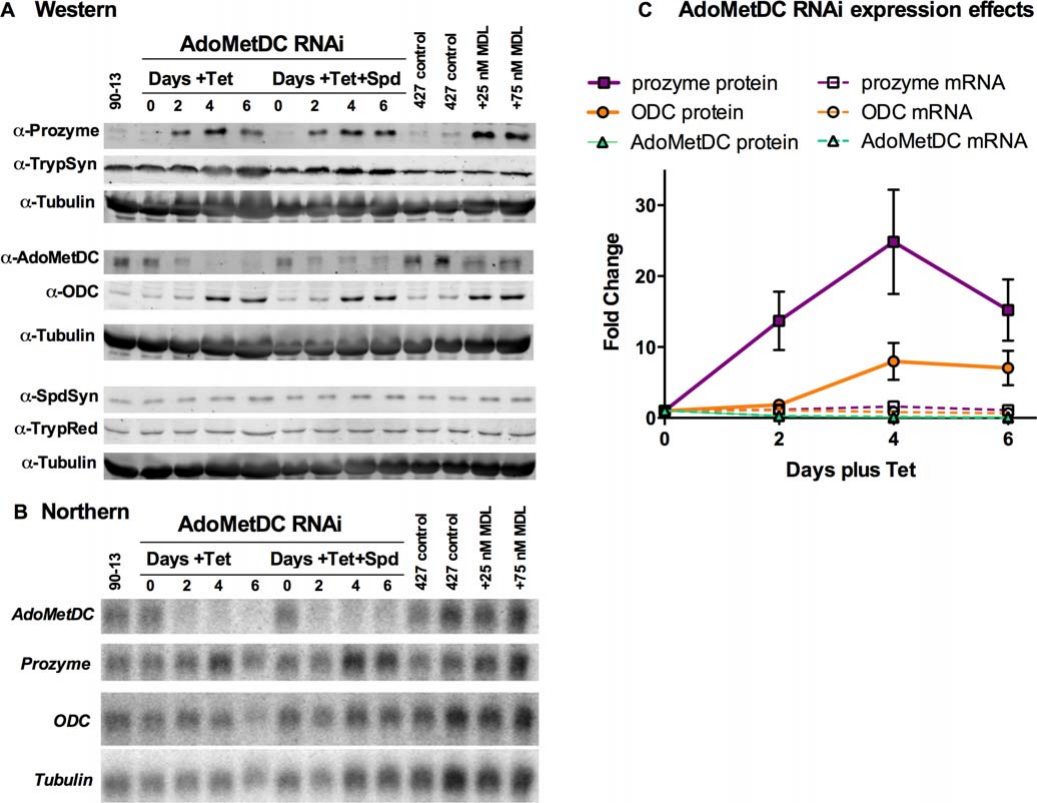
Comparison of prozyme and ODC protein and mRNA levels upon AdoMetDC knockdown. The AdoMetDC RNAi line was induced with Tet, and samples were collected over the time range and conditions described in Figure 2. A representative data set is displayed. Samples were visualized on a Typhoon 8610 scanner and quantitated as described in Materials and Methods. (A) Western analysis. (B) Northern blot analysis. (C) Quantitation of relative ODC and prozyme protein (solid symbols) and mRNA (open symbols) levels. Protein and mRNA levels were normalized to tubulin controls and are displayed as a fold change relative to uninduced control cells (−Tet). AdoMetDC, green triangles; prozyme, purple squares; and ODC, orange circles. The plotted data can be found in Tables S3 and S4. Errors represent the standard error of the mean.

We undertook quantitative analysis of the effects on protein and mRNA levels to gain mechanistic insight into the observed induction of prozyme and ODC upon knockdown of AdoMetDC or prozyme. Protein amounts were determined by quantitative Western blotting with fluorescent secondary antibodies, and Northern blots were developed by phosphorimaging; the density of these signals was then quantitated using imaging software and the effects determined relative to tubulin controls (Figure 5A and 5B and Table S3 show a representative data set). AdoMetDC protein and mRNA levels were reduced by 70–80% compared to controls by induction of RNAi, consistent with the activity data. In response, as observed in Figures 2 and 3, both prozyme and ODC protein levels are induced. The induction of prozyme is consistently more robust and occurs earlier in the time course than for ODC. Prozyme protein increased by an average of 25-fold (range 12–40-fold, n = 3), while ODC increased 7-fold (range 5–10-fold, n = 3) (day 4 +Tet; Figure 5C and Table S4). In the presence of spermidine, knockdown of AdoMetDC also led to induction of prozyme and ODC protein, with observed average increases of 10 and 5-fold, respectively (day 4 +Tet, +Spd). Finally, quantitative analysis of the prozyme cKO line showed that the loss of prozyme expression also led to induction of ODC, with ODC protein levels increasing by 4- and 5-fold (day 2 and 3 without Tet, respectively) (Figure S5 and Table S4).

In order to confirm that the magnitude of the ODC protein induction was accurately measured by our quantitative analysis we also performed activity assays on the same sample set. ODC activity increased between 8–13-fold (days 4 and 6 no Spd) and by 4–5 fold (days 4 and 6 plus Spd) after induction of AdoMetDC RNAi, and by 9-fold after the expression of prozyme was turned off in the cKO line (day 3 −Tet). These data show that the observed induction of ODC protein correlates with a similar increase in ODC activity.

For both prozyme and ODC the mRNA levels remained relatively constant, and were within 2-fold of the levels detected for control samples (Figure 5B and Table S3), thus the observed changes in protein expression arise predominately from increased protein levels. These data suggest that the expression of prozyme and ODC is controlled either at the level of translation, or through a post-translational mechanism. In contrast, the expression levels of the remaining pathway enzymes are not regulated under the experimental conditions and the protein and mRNA levels remain within 2-fold of the uninduced controls (Figure 5 and Table S3).

### Prozyme and ODC protein levels are induced by the AdoMetDC inhibitor MDL 73811

**Figure 6:**
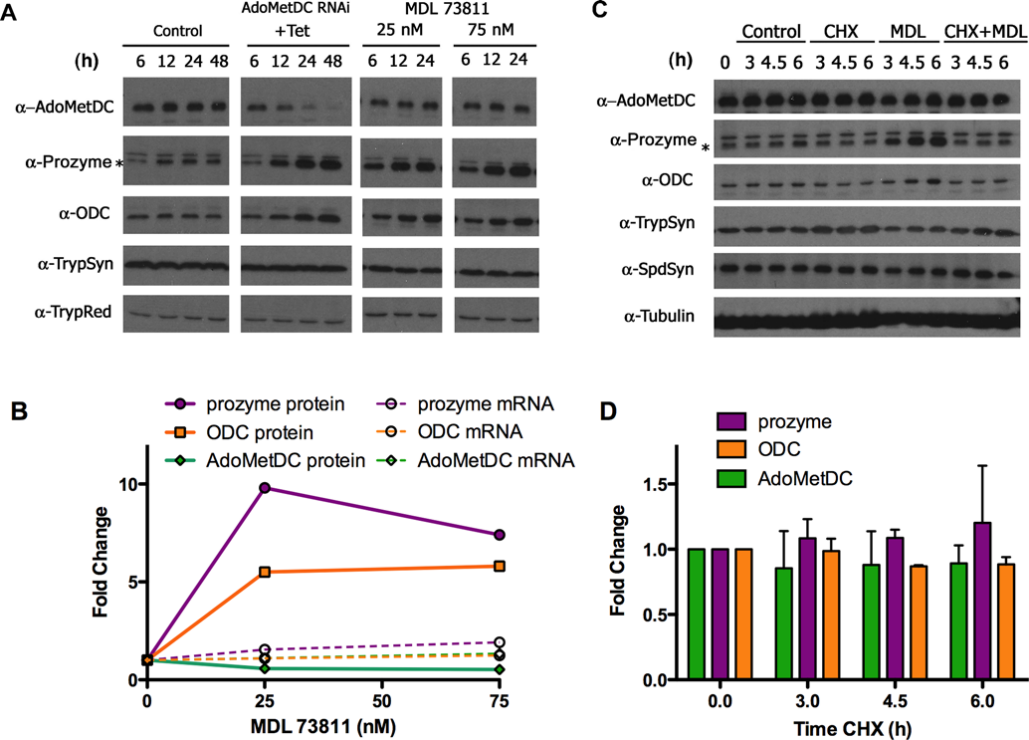
The effects of chemical inhibition of AdoMetDC on the expression of polyamine biosynthetic enzymes. (A) Comparison of AdoMetDC genetic knockdown with AdoMetDC inhibition by MDL 73811. AdoMetDC RNAi cells were split into fresh media and induced with Tet, or treated with 25 or 75 nM MDL73811. Cells were collected over a 24–48 h time course and analyzed by Western blot. (B) Analysis of protein and mRNA levels in 427 cells treated with MDL 73811 for 24 h. Protein (solid symbols) and mRNA (open symbols) were normalized to tubulin controls and then displayed as a fold change relative to untreated cells. The gel used for quantitation is displayed in Figure 5. AdoMetDC, green triangles; prozyme, purple squares; and ODC, orange circles. (C) The effect of cycloheximide treatment on protein expression. Cycloheximide (CHX) (50 µg/ml) and/or MDL 73811 (MDL) (75 nM) were added to the culture and cells were collected for Western analysis after 3, 4.5, and 6 hrs of drug treatment. Control cells underwent identical procedures but were not treated with either CHX or MDL73811. (D) Quantitation of protein levels of AdoMetDC, prozyme, and ODC during treatment with cycloheximide. Values are shown as the ratio to tubulin controls, normalized to untreated cells at time zero. AdoMetDC, green; prozyme, purple; and ODC, orange. Errors represent the standard error of the mean (n = 2).

In order to determine if the induction of prozyme and ODC in the AdoMetDC RNAi line was due to the loss of AdoMetDC protein or AdoMetDC activity, we monitored protein levels by Western blotting in the presence of MDL 73811 (5′-([(Z)-4-amino-2-butenyl]methylamino)-5′-deoxyadenosine), a suicide inhibitor of AdoMetDC [15]. The growth effects of MDL 73811 were first determined for blood form 427 parasites and for the AdoMetDC RNAi line in the absence of Tet (EC_50_ = 25–50 nM). Cells were then cultured in MDL 73811 concentrations near the EC_50_ (25 and 75 nM MDL 73811) and AdoMetDC, prozyme, ODC, TrypSyn and TrypRed were followed by Western over a 24–48 h time course (Figures 5, 6A, and 6B, and Table S4). The AdoMetDC RNAi cell line in the absence of MDL 73811 was followed as a control (±Tet). Knockdown of AdoMetDC by RNAi led to induction of prozyme and ODC protein within 12 h of Tet addition. Prozyme and ODC protein levels were induced by MDL 73811 as early as 3 h after its addition to the culture, with protein levels increasing by 10 and 6-fold, respectively after 24 h (Figures 5 and 6). AdoMetDC levels declined by 40% in the presence of MDL 73811, while the levels of TrypSyn, SpdSyn and TrpRed were unchanged. The mRNA levels of the pathway enzymes were within 2-fold of the levels for the control cells after MDL 73811 treatment. These data demonstrate that prozyme and ODC protein levels are rapidly upregulated by either genetic knockdown of AdoMetDC or by its chemical inhibition.

### Prozyme and ODC are stable in the presence of cycloheximide

The protein synthesis inhibitor cycloheximide was added to the cells to determine if changes in protein turnover rates account for the induction of prozyme and ODC protein upon loss of AdoMetDC activity. Cycloheximide prevented the induction of both prozyme and ODC by MDL 73811, demonstrating that new protein synthesis is required to observe the upregulation of both proteins. AdoMetDC, prozyme and ODC protein levels in the control cells were stable over the course of the 6 h experiment (Figure 6C and 6D), suggesting that changes in protein turnover rates do not account for the rapid induction of protein levels observed either in the presence of MDL 73811 or during genetic depletion of AdoMetDC. Therefore, changes in translational efficiency are implicated as the mechanism for induction of prozyme and ODC.

## Discussion

Polyamines are essential growth modulators that are highly regulated in eukaryotic cells. The studies herein demonstrate that AdoMetDC and prozyme are both essential for the growth of blood form *T. brucei*, and they provide the first evidence that the polyamine biosynthetic pathway is also regulated in *T. brucei*. Our data implicate both prozyme and trypanothione in regulation of polyamine homeostasis in *T. brucei*, suggesting that the evolution of these trypanosomatid-specific factors may have been linked to the need to acquire regulatory control mechanisms to modulate the growth effects of polyamines on the cell.

Few targets in the *T. brucei* parasite are validated both genetically and chemically, and the data presented here contribute to a compelling case that AdoMetDC is a promising drug target for the development of new treatments for African sleeping sickness. We have shown that prozyme is an essential activator of AdoMetDC activity and that the low activity of the AdoMetDC homodimer on its own is insufficient to maintain cell growth. Thus inhibitors that block heterodimerization, or lock the AdoMetDC structure into an inactive confirmation would provide a parasite-specific mechanism to inhibit this essential enzyme. Successful strategies for these approaches have recently been described, e.g. for Abl kinase [21] and the Bcl-X/BAK interaction [22]. The AdoMetDC knockdown by RNAi led to an 70–80% reduction in AdoMetDC protein that was sufficient to cause cell death, suggesting that a small molecule inhibitor of AdoMetDC would not need to fully inhibit AdoMetDC to be effective. A postulated mechanism for selective toxicity of ODC inhibitors is differential rates of intracellular protein turnover between the rapidly degraded mammalian ODC and the stable trypanosome enzyme [23]. We find that *T. brucei* AdoMetDC is also a stable protein, while like ODC mammalian AdoMetDC has a short intracellular half-life [24]. Thus differential enzyme turnover may also contribute to selective toxicity of AdoMetDC inhibitors in *T. brucei*. Finally, the AdoMetDC suicide inhibitor MDL 73811 is a potent anti-trypanosomal agent [14],[15]. Our finding that prozyme levels are induced by MDL 73811 provides strong evidence supporting AdoMetDC inhibition as the mechanism of action of MDL 73811 in *T. brucei*, strengthening the chemical validation of the target.

It appears that *T. brucei* regulates the polyamine pathway flux to maintain spermidine homeostasis while depleting the conjugated thiol pools. Indeed spermidine levels do not fall below 40% of the control levels after induction of AdoMetDC RNAi, and they are only fully depleted in the prozyme cKO, which generated a more complete knockdown of AdoMetDC activity. Addition of exogenous spermidine to the prozyme cKO line partially restored the spermidine pools, but not the GSH-Spd and trypanothione pools, suggesting that the cell does not build up the GSH-Spd and trypanothione pools until spermidine levels reach an appropriate steady-state set point. In support, eflornithine treatment has been reported to cause only a partial depletion of spermidine [25],[26]. These data further provide evidence that the loss of trypanothione pools is a central factor in cell death for both the AdoMetDC knockdown cells and during eflornithine treatment. TrypSyn contains both a synthetic domain catalyzing the formation of GSH-Spd and trypanothione, as well as a catabolic domain that is able to degrade both conjugates back to free spermidine and GSH [27]. The function of the catabolic domain has not been demonstrated *in vivo*, however our data supports the idea that trypanothione serves as a cellular reservoir for spermidine and that its catabolism contributes to spermidine homeostasis in the cell. This mechanism would provide an analogous situation to that observed in mammalian cells where catabolism of spermine and spermidine play crucial roles in regulating pathway flux [28].

The finding that spermidine was able to rescue the cell growth defect caused by the AdoMetDC RNAi knockdown, but not the prozyme cKO is not entirely understood. Underlying this observation is the finding that exogenous spermidine restored the spermidine and trypanothione pools to the AdoMetDC RNAi cells but not the prozyme cKO cells. We did not observe any difference in spermidine uptake between these lines (unpublished observation) but did find as has been previously published that spermidine is poorly taken up by *T. brucei* blood form parasites [10]. The prozyme cKO led to a 30% greater reduction in AdoMetDC activity than the AdoMetDC RNAi knockdown, and this correlates with a more rapid and complete depletion of spermidine and trypanothione and to more rapid cell growth arrest. This data, in combination with the spermidine pool analysis, suggests that spermidine incorporation is only sufficient to restore GSH-Spd and trypanothione pools after AdoMetDC RNAi, where more residual AdoMetDC remains to help bolster the spermidine pools than is observed in the prozyme cKO (Figure 4). A further caveat is the observation that spermidine is able to rescue the AdoMetDC RNAi lines when added up to day 4 after Tet induction, while it can not rescue these lines if added as late as day 6. This corresponds to the point in the growth curve where the GSH-Spd and trypanothione pools have become completely depleted, suggesting that the cells have undergone an irreversible event in the absence of these pools that commits them to death. For the prozyme cKO line, even at the earliest time point measured (day 2), the GSH-Spd and trypanothione pools were completely depleted, so it may be that the prozyme cKO cells undergo the irreversible transition too quickly for spermidine rescue to be effective.

*T. brucei* responds to loss of spermidine by upregulating two key proteins required for spermidine production, ODC and prozyme. Either the genetic knockdown of AdoMetDC or its chemical inhibition by MDL 73811 led to a rapid and robust increase in prozyme and ODC protein levels. Spermidine rescue of the AdoMetDC RNAi line fully restored normal cell growth, yet the induction of the prozyme and ODC proteins still occurred, providing strong evidence that the induction was not linked to non-specific cell growth effects. The fact that either genetic or chemical loss of AdoMetDC activity leads to increased protein levels of ODC and prozyme provides cross-validation for the observation and demonstrates that the effects do not result from some peculiarity of the genetic system. In wild-type cells prozyme is present in limiting concentration in comparison to AdoMetDC. Thus prozyme is positioned to function not only as an essential activator of AdoMetDC, but by dynamically altering its levels the cell has a mechanism to modulate spermidine production depending on its metabolic state. The pyruvoyl-dependant mechanism of AdoMet decarboxylation is inherently prone to enzyme inactivation through abortive deamination, which occurs at a significant frequency during the catalytic cycle [29]. Mammalian cells may utilize rapid turnover of AdoMetDC as a mechanism to replace the damaged enzyme. Perhaps in the trypanosomatids, where AdoMetDC is a stable protein, the ability to rapidly upregulate the allosteric activator, prozyme, provides an alternative mechanism to evade substrate-mediated inactivation of AdoMetDC by allowing the AdoMetDC pools to remain inactive until needed.

The expression of prozyme and ODC both appear to be under translational control. First the protein levels of prozyme and ODC increased substantially upon loss of AdoMetDC activity, while the mRNA levels were not significantly affected. Thus the increased protein accumulation results from either increased translational or from post-translational changes in protein stability. Secondly, prozyme, ODC and AdoMetDC were stable over the 6 h incubation with cycloheximide demonstrating that changes in protein turnover are unlikely to contribute to the increase in steady-state protein levels. The control of gene expression in *T. brucei* is unusual in comparison to other eukaryotic cells. In *T. brucei* mRNAs are transcribed as polycistronic units and polymerase II gene transcription is not regulated at the level of transcription initiation [19]. Instead gene expression is regulated post-transcriptionally through changes in message stability, protein stability or through translational regulation. Examples of translational control in *T. brucei* are limited but include developmental regulation of procyclin [30]–[32], cytochrome oxidase isoforms [33] and of Nrk protein kinase [34]. Cytochrome c has been shown to be regulated post-translationally by differential protein stability [35]. The finding that polyamine biosynthesis in *T. brucei* is likely to be translationally regulated shows that translational control in *T. brucei* is not limited to developmental regulation but also functions as a mechanism to regulate house-keeping enzymes involved in primary metabolism during the cell cycle.

An open question is what triggers the de-repression of ODC and prozyme protein expression in response to loss of AdoMetDC. Protein levels are increased by either knockdown or inhibition of AdoMetDC, thus loss of AdoMetDC activity appears to be the common element in triggering the response. The precedent in other eukaryotic cells is that changes in spermidine levels affect the ribosome and thus influence translation. Expression of the ODC inhibitor antizyme is controlled by ribosomal frame-shifting which is stimulated by spermidine, or by putrescine at higher concentrations [36],[37]. Translation of AdoMetDC in mammalian cells and plants is regulated by a ribosome-stalling peptide that traps the ribosome so that AdoMetDC mRNA is translated efficiently only at low spermidine concentration [38],[39]. In *T. brucei* there are no apparent upstream open reading frames in prozyme or ODC, and changes in spermidine concentration appear unlikely to trigger the observed translational changes, since protein levels were induced even in the spermidine rescued AdoMetDC RNAi knockdown cells. The elevated putrescine levels (6–10-fold) observed in the metabolite profiles for cells that express higher levels of prozyme and ODC is a potentially significant observation, but it seems an unlikely trigger for an increase in ODC expression. Human and malaria dihydrofolate reductase have been shown to bind their mRNA to regulate translation of the message [40],[41]. For the human enzyme binding to mRNA is prevented by the enzyme inhibitor methotrexate. This suggests an intriguing hypothesis that is consistent with our data. AdoMetDC may bind to both the prozyme and ODC mRNA and thereby inhibit their translation.

The regulated expression of prozyme is a unique mechanism for controlling polyamine pathway flux in *T. brucei* not found in other eukaryotic cells. However, despite differences in the protein components the metabolic control points of the pathway in *T. brucei* are similar to those found in mammalian cells, with regulation being focused on control of ODC and AdoMetDC. Thus the data suggests that similarly to mammalian cells, ODC and AdoMetDC catalyze the rate-limiting steps in polyamine biosynthesis in *T. brucei*. While the mechanisms differ, both utilize translational regulation of regulatory proteins to control the pathway flux. Mammalian cells translationally regulate an ODC inhibitor protein, antizyme, while *T. brucei* regulates an AdoMetDC activator protein, prozyme. Thus *T. brucei* has evolved a unique but parallel regulatory mechanism to control polyamine metabolism to that found in mammalian cells. These data provide evidence for convergent evolution of translational control mechanisms centered on regulatory binding proteins in the polyamine metabolic pathway.

## Materials and Methods

### Trypanosome culture

Bloodstream form trypanosomes (90-13 or 427) were cultured in HMI-9 media supplemented with 10% serum at 37°C, 5% CO_2_ as described [42]. Chicken serum was used in place of fetal bovine serum, allowing for the addition of spermidine (0.1 mM) without encountering polyamine oxidase-driven toxicity [7]. Cells were grown with the appropriate antibiotics (G418, 2.5 µg/ml; hygromycin 5 µg/ml; phleomycin 2.5 µg/ml, blasticidin 2.5 µg/ml) and were split every 24–48 hours to maintain cultures in log phase (10^5^ to 10^6^ cells/ml). Cell densities were determined by counting on a hemocytometer (Brightline, Fisher). Growth curves are represented as total cell number (product of cell density and total dilution) and all data were collected in biological triplicate.

To determine the effects of MDL 73811, AdoMetDC RNAi or 427 cells were cultured in the in the presence of a range of MDL 73811 concentrations (10–150 nM MDL 73811) and cells were counted after 3 d to determine the EC_50_. For Western analysis cultures of blood form *T. brucei* wild-type cells were split into fresh media 3 h before dosing with inhibitors. Drug (25 or 75 nM), cycloheximide (50 µg/ml) or both were then added and samples were collected at the indicated time points. The ability of this concentration of cycloheximide to inhibit protein synthesis in *T. brucei* has previously been established [23],[33],[43].

### Preparation of AdoMetDC RNAi constructs and cell lines

The techniques for manipulating gene expression in *T. brucei* by RNAi have been well established [44]. The pLEW100 and pJM326 vectors [45] were used to generate the AdoMetDC RNAi plasmid. A 620 base pair portion of the *T. brucei* AdoMetDC gene (starting at the 78^th^ coding nucleotide) was amplified by PCR (primers in Table S5) from genomic DNA isolated from *T. brucei* 427 cells and cloned into the pJM326 vector in the forward direction and into the pLew100 vector in the reverse direction. The AdoMetDC fragment fused to the stuffer region was excised from pJM326 with HindIII and XbaI and inserted into the modified pLew100 vector. The resulting plasmid contains two copies of the AdoMetDC gene fragment in opposite orientation separated by the stuffer DNA sequence (Figure S1A). The production of stemloop RNA (with double stranded RNA targeting the AdoMetDC message) is driven from the Tet inducible procyclin promoter.

Log phase *T. brucei* 90-13 bloodstream form cells were transfected with the linearized AdoMetDC stemloop RNAi vector (80 µg), and phleomycin resistant cells containing the construct integrated into the rRNA locus were selected as previously described [11]. Clonal lines were generated by limiting dilution. Synthesis of double stranded RNA (RNAi) targeting the AdoMetDC message in a stemloop structure was induced by Tet (1 µg/ml), which was added fresh every 24 h.

### Preparation of prozyme cKO constructs and cell lines

The prozyme conditional knockout line was generated using previously described methods [46],[47]. Identical 300 bp segments of the prozyme 5′ and 3′ UTRs (corresponding to nucleotide −321 to −1 and 1023 to 1348, respectively where base 1 represents the ATG start and base 978 the TGA stop codons in the protein coding region) were cloned into the pLEW13 and pLEW90 vectors. The pLEW100 vector was altered by liberating the phleomycin resistance cassette with DraIII and BssHII and replacing it with the blasticidin resistance gene from pcDNA6V5His (Invitrogen). The resulting small open reading frame prior to the blasticidin coding region was removed by site directed mutagenesis. The resulting vector (pLEW300) was used to generate the Tet inducible expression plasmid containing Flag-tagged prozyme, using the tagged gene isolated from the previously described pT7-FLAG1-prozyme vector [20]. The primers used to generate the constructs are provided in Table S5, and the construct diagrams are in Figure S1B.

Log phase *T. brucei* 427 bloodsteam form cells were transfected with the Amaxa nucleofector as described [48] to introduce in successive steps: 1) the pLew13-prozyme SKO-N vector (which contains coding sequence for T7 polymerase and the G418 resistance cassette inside the prozyme 5′ and 3′ UTRs) integrated into the prozyme locus; 2) the pLew300-Flag prozyme construct (which contains coding sequence for a FLAG tagged prozyme and the blasticidin resistance cassette) targeted to the rDNA spacer region; and, 3) pLew90-prozyme SKO-H vector (which contains coding sequence for the tet repressor and the hygromycin resistance cassette inside the prozyme 5′ and 3′ UTRs) integrated into the second prozyme locus. Clonal lines were generated by limiting dilution. Prozyme expression was maintained in these lines by the addition of Tet (1 µg/ml).

### Preparation of genomic DNA and Southern blot analysis

Southern blot analysis was used to confirm the genotypes of cKO cell lines. Genomic DNA (10 µg) was isolated from cells using standard methods, digested with BanI, separated on a 1% agarose gel and transferred to a positively charged membrane (Ambion Bright Star) by vacuum in 10× SSC. The membrane was crosslinked by UV, prehybridized in Ambion UltraHyb solution and then hybridized with probe overnight. Blots were washed (0.1× SSC and 0.1% SDS) and visualized with a FLA 5000 phosphoimager. Radiolabeled [^32^P]dATP probes were prepared using the Strip-EZ PCR kit (Ambion) from genomic DNA. The primers used for generation of the probe by PCR are provided in Table S5. The probe for the endogenous locus was a 600 bp region starting at 891 bp upstream of the prozyme ATG start site. The probe for the coding region included nucleotides 78 to 678 of the prozyme gene.

### Western blot analysis

Cells (∼10^8^) were harvested by centrifugation (3,000 rpm), washed twice in cold phosphate-buffered saline (PBS, pH = 7.4), resuspended in lysis buffer (50 mM HEPES pH 8, 100 mM NaCl, 5 mM 2-mercaptoethanol, 2 mM phenylmethylsulfonyl fluoride, 1 µg/ml leupeptin, 2 µg/ml antipain, 10 µg/ml benzamidine, 1 µg/ml pepstatin, 1 µg/ml chymostatin), and lysed by three successive freeze/thaw cycles. The lysate was clarified by centrifugation (13,200 rpm for 5 minutes) and protein concentration was determined colormetrically [49].

Protein (20 µg/lane) was separated by 12% SDS/PAGE and transferred to a polyvinylidene difluoride (PVDF) membrane (Hybond-P, Amersham). Membranes were blocked in 5% non-fat milk in Tris-buffered saline (TBS: 20 mM Tris·HCl, 137 mM NaCl, pH 7.6). Primary and secondary antibody incubations were carried out in 5% milk TBS-T (TBS plus 0.1% (v/v) Tween-20). Rabbit polyclonal antibody against *T. brucei* prozyme was generated (by Proteintech Group, Inc, Chicago, IL) to recombinant His_6_-prozyme purified as described [20] and was used at 4,000-fold dilution. All other antibodies have been previously described [8],[11],[12],[20] and were used at the following dilutions: *T. brucei* AdoMetDC (2,500-fold dilution), ODC (10,000-fold dilution), *L. donovani* SpdSyn (1000-fold dilution), *T. brucei* TrypSyn (1,000-fold dilution) and *T. cruzi* TrypRed (1000-fold dilution). Mouse anti-FLAG M2 antibody (Sigma)(1,000-fold dilution), horseradish peroxidase-(HRP) linked donkey anti-rabbit or anti-mouse IgG secondary antibodies (Amersham Biosciences) were used at 10,000 fold dilution. Antigen recognition was visualized using the ECL chemiluminescent HRP substrate reagents (Amersham), followed by detection on film. For the AdoMetDC RNAi cells (Figure 2), separate gels were prepared for each antibody and the full molecular weight range was transferred to membrane and probed. This strategy allowed any potential modifications of the proteins to be detected, and none were observed. Thus only the appropriate molecular weight range for the probed protein is displayed in Figure 1. For the prozyme cKO cells (Figure 3) parallel gels were loaded and transferred, but each membrane was sectioned into three strips by molecular weight to allow for probing of three antibodies from one gel. Western analysis was repeated in biological triplicates and representative experimental sets are displayed (Figures 2B and 3B).

### Quantitative Western analysis

Samples were prepared as described above, parallel gels were loaded, and each gel was cut into three sections to allow for probing of multiple antibodies. Each gel was probed for tubulin as a control. mRNA from these samples was also collected and used for quantitative northern blot analysis (see below). Primary antibody raised against AdoMetDC was used at a 500-fold dilution, tubulin was used at 50,000-fold dilution, and all other antibodies were used at a 1000-fold dilution in 5% milk-TBS-T. Alexa-Fluor 647 goat anti-rabbit IgG, Alexa-Fluor 647 goat anti-mouse IgG or Alexa-Fluor 546 goat anti-mouse IgG (Invitrogen) secondary antibodies were used at a 500-fold (for AdoMetDC blot) or 1,000-fold (all other antibodies) in 5% BSA (bovine serum albumin) TBS-T. Fluorescent signals were measured on a Typhoon 8610 (Molecular Dynamics,with λ_ex_ = 633 and λ_em_ = 670 or λ_ex_ = 532 and λ_em_ = 580). The bands were quantified by ImageQuant 5.2 software (Molecular Dynamics). Density from each antibody was normalized by tubulin density, and each experimental condition was normalized to control samples.

### Quantification of intracellular polyamine and thiol pools

Intracellular polyamine content was analyzed by conjugation to the fluorescent AccQ-tag reagent (6-aminoquinolyl-*n*-hydroxysuccinimidyl in acetonitrile, Waters) followed by separation on a Waters AccQtag (3.9×150 mm) column using a Beckman System Gold HPLC with a Ranin Dynamax Fluorescence detector. HPLC buffers and gradients have been previously described [50]. The reduced intracellular thiols were quantitated as described [11],[26] by labeling with monobromobimane and separation on a Phenomonex Nucleosil C_18_ column (30×4.6 mm).

### Enzymatic assays of AdoMetDC and ODC activity in cell lysates

AdoMetDC and ODC activity in cell lysates were determined as previously described [20],[50]. Lysates (40 µg) were incubated with ^14^C-AdoMet (Amersham; 40 µM) ± recombinant prozyme (1 µM) for assay of AdoMetDC or with 1-^14^C-ornithine (Amersham; 80 µM) for assay of ODC. Reactions were allowed to proceed for 30 minutes at 37°C before quenching with HCl. Liberated ^14^CO_2_ was trapped by barium hydroxide soaked filter paper and measured by scintillation counting.

### Northern analysis

Northern blot analysis was facilitated by the NorthernMax kit (Ambion). Briefly, mRNA was isolated from trypanosome cells (at least 1×10^8^ ) using the micro polyA purist kit (Ambion) and separated by denaturing 1% agarose gel electrophoresis (1 µg/lane). The mRNA was transferred to a positively charged nylon membrane (BrightStar-Plus, Ambion) and cross-linked. Radiolabeled [^32^P]dATP probes were prepared using the Strip-EZ PCR kit (Ambion) with genomic or plasmid DNA serving as the template. Data displayed represents a single membrane that was stripped and reprobed for each of the displayed genes. Signals were either developed on film, or with a phosphorimager screen. For quantitation, the signal from the phosphorimager screen (Molecular Dynamics) was measured on the Typhoon 8610 scanner (Molecular Dynamics, using the 633 nm laser), and quantified my ImageQuant 5.2 software (Molecular Dynamics).

### Gene accession numbers

Gene accession numbers have been taken from http://www.genedb.org/ and are as follows: AdoMetDC, Tb927.6.4460/Tb927.6.4410; prozyme, Tb927.6.4470; ODC, Tb11.01.5300; Spd Syn, Tb09.v1.0380; Tryp Syn, Tb927.2.4370 and Tryp Red, Tb10.406.0520.

## Supporting Information

Figure S1 RNAi and cKO vector diagrams. (A) Vector used to generate the AdoMetDC stem-loop RNAi construct. (B) Vectors used to create the prozyme conditional knockout line. Vectors were inserted into blood form parasites in three steps, starting with vector 1. Vector construction and trypanosome transfection are described in the Materials and Methods section.
(1.99 MB TIF)

Figure S2 AdoMetDC RNAi cells can be rescued up to four days after RNAi induction. Spermidine was added to AdoMetDC RNAi cultures at the time of Tet addition, or two four or six days post induction, and growth of the cells was monitored.
(17.94 MB TIF)

Figure S3 Tet treatment of blood form *T. brucei* control cells does not lead to prozyme or ODC induction. Untransfected 90-13 and 427 bloodstream form cells were cultured in the presence and absence of Tet for four days.
(4.73 MB TIF)

Figure S4 Southern blot confirmation of prozyme cKO. Genomic DNA was harvested from parental 427, single knockout SKO-N (with integrated K.O. vector 1), SKO-H (with integrated K.O. vector 3), the precursor SKO-N-FP (integrated K.O. vector 1 and exogenous FLAG-prozyme vector 2) and the prozyme cKO (integrated K.O. vector 1, integrated K.O vector 3 and exogenous FLAG-prozyme vector 2) cell lines. The genomic DNA was digested with Ban1 and probed with a region of the 5′UTR (indicated with the blue line) that is present in the endogenous locus and in each knockout vector (1 and 3, see Figure S1B). Locus representation is not to scale.
(11.70 MB TIF)

Figure S5 mRNA and protein levels in AdoMetDC RNAi prozyme cKO cells. Northern blot analysis of mRNA from AdoMetDC RNAi (A) or prozyme cKO (B) and Western analysis of prozyme cKO (C) cells in the conditions described in Figures 2 and 3. For Northern analysis, data for each set represent the results from a single gel that was stripped and reprobed for each gene.
(5.95 MB TIF)

Table S1 Metabolite Analysis of AdoMetDC RNAi cells.
(0.04 MB DOC)

Table S2 Metabolite Analysis of prozyme cKO cells.
(0.04 MB DOC)

Table S3 Quantification of protein and mRNA levels. (A) Normalized ratio of protein to tubulin. AdoMetDC RNAi and 90-13 parental cells were normalized to the uninduced controls grown in the same condition. MDL 73811 treated cells were normalized to the average of untreated 427 controls collected at the identical time point (24 hours). (B) Normalized ratio of mRNA to tubulin. Data were analyzed as in (A). (C) Normalized ratios of protein to mRNA. Ratios of the values for protein/tubulin to mRNA/tubulin are displayed.
(0.06 MB DOC)

Table S4 Protein Induction: Fold change over controls. AdoMetDC RNAi (n = 3) and Prozyme cKO samples (n = 2) were analyzed by quantitative Western as described in the manuscript. Reported error represents the standard error of the mean. Tet was added (AdoMetDC RNAi) or removed (prozyme cKO) on day (D) zero, samples were collected on D2–D6.
(0.03 MB DOC)

Table S5 Oligonucleotide primers.
(0.03 MB DOC)

## References

[1] WHO (2007). Fact sheet on African trypanosomiasis.. http://www.who.int/mediacentre/factsheets/fs259/en/.

[2] Barrett MP, Boykin DW, Brun R, Tidwell RR (2007). Human African trypanosomiasis: pharmacological re-engagement with a neglected disease.. Br J Pharmacol.

[3] Pegg AE, Feith DJ (2007). Polyamines and neoplastic growth.. Biochem Soc Trans.

[4] Casero RA, Marton LJ (2007). Targeting polyamine metabolism and function in cancer and other hyperproliferative diseases.. Nat Rev Drug Discov.

[5] Krauth-Siegel LR, Comini MA, Schlecker T (2007). The trypanothione system.. Subcell Biochem.

[6] Jiang Y, Roberts SC, Jardin A, Carter NS, Shih S (1999). Ornithine decarboxylase gene deletion mutants of *Leishmania donovani*.. J Biol Chem.

[7] Roberts SC, Scott J, Gasteier JE, Jiang Y, Brooks B (2002). S-adenosylmethionine decarboxylase from *Leishmania donovani*: molecular, genetic and biochemical characterization of null mutants and overproducers.. J Biol Chem.

[8] Roberts SC, Jiang Y, Jardim A, Carter NS, Heby O (2001). Genetic analysis of spermidine synthase from *Leishmania donovani*.. Mol Biochem Parasitol.

[9] Li F, Hua SB, Wang CC, Gottesdiener KM (1998). *Trypanosoma brucei brucei*: characterization of an ODC null bloodstream form mutant and the action of alpha-difluoromethylornithine.. Exp Parasitiol.

[10] Taylor MC, Kaur H, Blessington B, Kelly JM, Wilkinson SR (2008). Validation of spermidine synthase as a drug target in African trypanosomes.. Biochem J.

[11] Huynh TT, Huynh VT, Harmon MA, Phillips MA (2003). Gene knockdown of g-glutamylcysteine synthetase by RNAi in the parasitic protozoa *Trypanosoma brucei* demonstrates that it is an essential enzyme.. J Biol Chem.

[12] Ariyanayagam MR, Oza SL, Guther MLS, Fairlamb AH (2005). Phenotypic analysis of trypanothione synthetase knockdown in the African trypanosome.. Biochem J.

[13] Krieger S, Schwarz W, Ariyanayagam MR, Fairlamb AH, Krauth-Siegel RL (2000). Trypanosomes lacking trypanothione reductase are avirulent and show increased sensitivity to oxidative stress.. Molecular Microbiology.

[14] Bacchi CJ, Brun R, Croft SL, Alicea K, Buhler Y (1996). In vivo trypanocidal activities of new S-adenosylmethionine decarboxylase inhibitors.. Antimicrobial Agents and Chemotherapy.

[15] Bitonti AJ, Byers TL, Bush TL, Casara PJ, Bacchi CJ (1990). Cure of *Trypanosoma brucei brucei* and *Trypanosoma brucei rhodesiense* Infections in Mice with an Irreversible Inhibitor of S-Adenosylmethionine Decarboxylase.. Antimicrobial Agents and Chemotherapy.

[16] Pegg AE, Xiong H, Feith DJ, Shantz LM (1998). S-adenosylmethionine decarboxylase: structure, function and regulation by polyamines.. Biochem Soc Trans.

[17] Pegg AE (2006). Regulation of ornithine decarboxylase.. J Biol Chem.

[18] Coffino P (2001). Regulation of cellular polyamines by antizyme.. Nature Reviews Molecular Cell Biology.

[19] Clayton C, Shapira M (2007). Post-transcriptional regulation of gene expression in trypanosomes and leishmanias.. Mol Biochem Parasitol.

[20] Willert EK, Fitzpatrick R, Phillips MA (2007). Allosteric regulation of an essential trypanosome polyamine biosynthetic enzyme by a catalytically dead homolog.. Proc Natl Acad Sci USA.

[21] Noble ME, Endicott JA, Johnson LN (2004). Protein kinase inhibitors: insights into drug design from structure.. Science.

[22] Wells JA, McClendon CL (2007). Reaching for high-hanging fruit in drug discovery at protein-protein interfaces.. Nature.

[23] Phillips MA, Coffino P, Wang CC (1987). Cloning and Sequencing of the Ornithine Decarboxylase Gene from *Trypanosoma brucei*. Implications for enzyme turnover and selective a-difluoromethylornithine inhibition.. J Biol Chem.

[24] Pegg AE (1979). Investigation of the Turnover of Rat Liver S-adenosylmethionine Decarboxylase Using a Specific Antibody.. J Biol Chem.

[25] Bacchi CJ, Garofalo J, Mockenhaupt D, McCann PP, Diekema KA (1983). In vivo effects of alpha-DL-difluoromethylornithine on the metabolism and morphology of *Trypanosoma brucei brucei*.. Mol Biochem Parasitol.

[26] Fairlamb AH, Henderson GB, Bacchi CJ, Cerami A (1987). In vivo effects of difluoromethylornithine on trypanothione and polyamine levels in bloodstream forms of *T. brucei*.. Mol Biochem Parasitol.

[27] Fyfe PK, Oza SL, Fairlamb AH, Hunter WN (2008). Leishmania trypanothione synthetase-amidase structure reveals a basis for regulation of conflicting synthetic and hydrolytic activities.. J Biol Chem.

[28] Wang Y, Casero RA (2006). Mammalian polyamine catabolism: a therapeutic target, a pathological problem, or both?. J Biochem.

[29] Anton DL, Kutny R (1987). Mechanism of substrate inactivation of *Escherichia coli* S-adenosylmethionine decarboxylase.. Biochemistry.

[30] Urwyler S, Vassella E, Van Den Abbeele J, Renggli CK, Blundell P (2005). Expression of procyclin mRNAs during cyclical transmission of *Trypanosoma brucei*.. PLoS Pathog.

[31] Schurch N, Furger A, Kurath U, Roditi I (1997). Contributions of the procyclin 3′ untranslated region and coding region to the regulation of expression in bloodstream forms of *Trypanosoma brucei*.. Mol Biochem Parasitol.

[32] Clayton CE, Hotz HR (1996). Post-transcriptional control of PARP gene expression.. Mol Biochem Parasitol.

[33] Mayho M, Fenn K, Craddy P, Crosthwaite S, Matthews K (2006). Post-transcriptional control of nuclear-encoded cytochrome oxidase subunits in *Trypanosoma brucei*: evidence for genome-wide conservation of life-cycle stage-specific regulatory elements.. Nucleic Acids Res.

[34] Gale M, Carter V, Parsons M (1994). Translational control mediates the developmental regulation of the *Trypanosoma brucei* Nrk protein kinase.. J Biol Chem.

[35] Torri AF, Bertrand KI, Hajduk SL (1993). Protein stability regulates the expression of cytochrome c during the developmental cycle of *Trypanosoma brucei*.. Mol Biochem Parasitol.

[36] Matsufuji S, Matsufuji T, Miyazaki Y, Murakami Y, Atkins JF (1995). Autoregulatory frameshifting in decoding mammalian ornithine decarboxylase antizyme.. Cell.

[37] Rom E, Kahana C (1994). Polyamines regulate the expression of ornithine decarboxylase antizyme in vitro by inducing ribosomal frame-shifting.. Proc Natl Acad Sci.

[38] Hanfrey C, Elliott KA, Franceschetti M, Mayer MJ, Illingworth C (2005). A dual upstream open reading frame-based autoregulatory circuit controlling polyamine-responsive translation.. J Biol Chem.

[39] Law GL, Raney A, Heusner C, Morris DR (2001). Polyamine regulation of ribosome pausing at the upstream open reading frame of S-adenosylmethionine decarboxylase.. J Biol Chem.

[40] Ercikan-Abali EA, Banerjee D, Waltham MC, Skacel N, Scotto KW (1997). Dihydrofolate reductase protein inhibits its own translation by binding to dihydrofolate reductase mRNA sequences within the coding region.. Biochemistry.

[41] Zhang K, Rathod PK (2002). Divergent regulation of dihydrofolate reductase between malaria parasite and human host.. Science.

[42] Hirumi H, Hirumi K (1989). Continuous cultivation of *Trypanosoma brucei* blood stream forms in a medium containing a low concentration of serum protein without feeder cell layers.. J Parasitol.

[43] Fluck C, Salomone JY, Kurath U, Roditi I (2003). Cycloheximide-mediated accumulation of transcripts from a procyclin expression site depends on the intergenic region.. Mol Biochem Parasitol.

[44] Ullu E, Tschudi C, Chakraborty T (2004). RNA interference in protozoan parasites.. Cell Microbiol.

[45] Wang Z, Morris JC, Drew ME, Englund PT (2000). Inhibition of *Trypanosoma brucei* gene expression by rna interference using an integratable vector with opposing T7 promoters.. J Biol Chem.

[46] Schnaufer A, Panigrahi AK, Panicucci B, Igo RP, Wirtz E (2001). An RNA ligase essential for RNA editing and survival of the bloodstream form of *Trypanosoma brucei*.. Science.

[47] Wirtz E, Leal S, Ochatt C, Cross GA (1999). A tightly regulated inducible expression system for conditional gene knock-outs and dominant-negative genetics in *T. brucei.*. Mol Biochem Parasitol.

[48] Burkard G, Fragoso CM, Roditi I (2007). Highly efficient stable transformation of bloodstream forms of *Trypanosoma brucei.*. Mol Biochem Parasitol.

[49] Bradford MM (1976). A rapid and sensitive method for the quantitation of microgram quantities of protein utilizing the principle of protein-dye binding.. Anal Biochem.

[50] Osterman AL, Brooks HB, Jackson L, Abbott JJ, Phillips MA (1999). Lys-69 plays a key role in catalysis by *T. brucei* ornithine decarboxylase through acceleration of the substrate binding, decarboxylation and product release steps.. Biochemistry.

