# Supplementary figures and images for: Regulated Expression of an Essential Allosteric Activator of Polyamine Biosynthesis in African Trypanosomes
Source: PLoS Pathog. 2008 Oct 24;4(10):e1000183. doi: 10.1371/journal.ppat.1000183 (PMC2562514; doi:10.1371/journal.ppat.1000183)

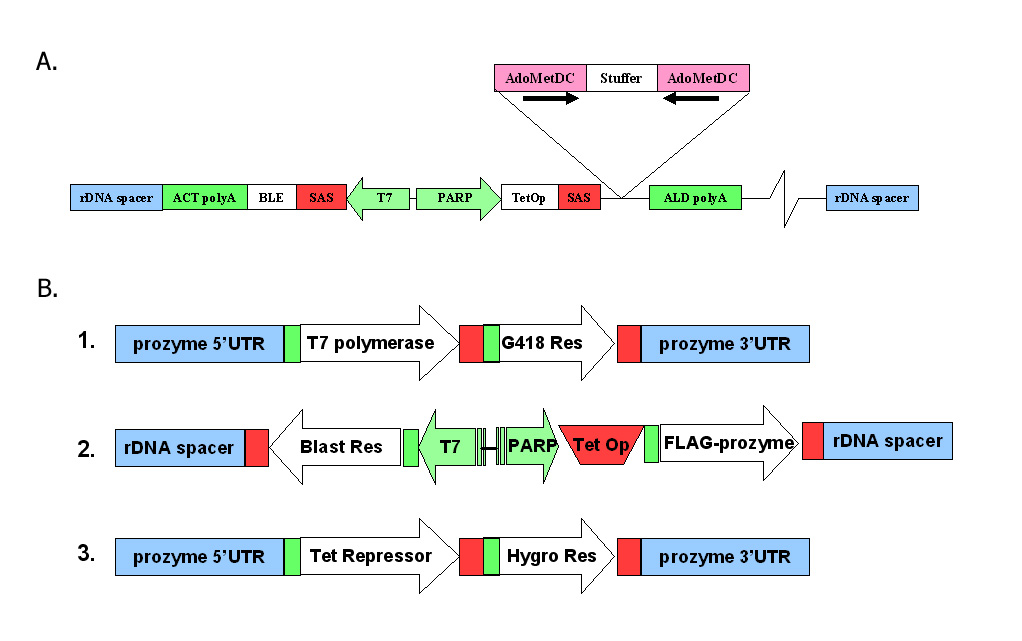

Supplement: Figure S1 — RNAi and cKO vector diagrams. (A) Vector used to generate the AdoMetDC stem-loop RNAi construct. (B) Vectors used to create the prozyme conditional knockout line. Vectors were inserted into blood form parasites in three steps, starting with vector 1. Vector construction and trypanosome transfection are described in the Materials and Methods section. (1.99 MB TIF) [file ppat.1000183.s001.tif]

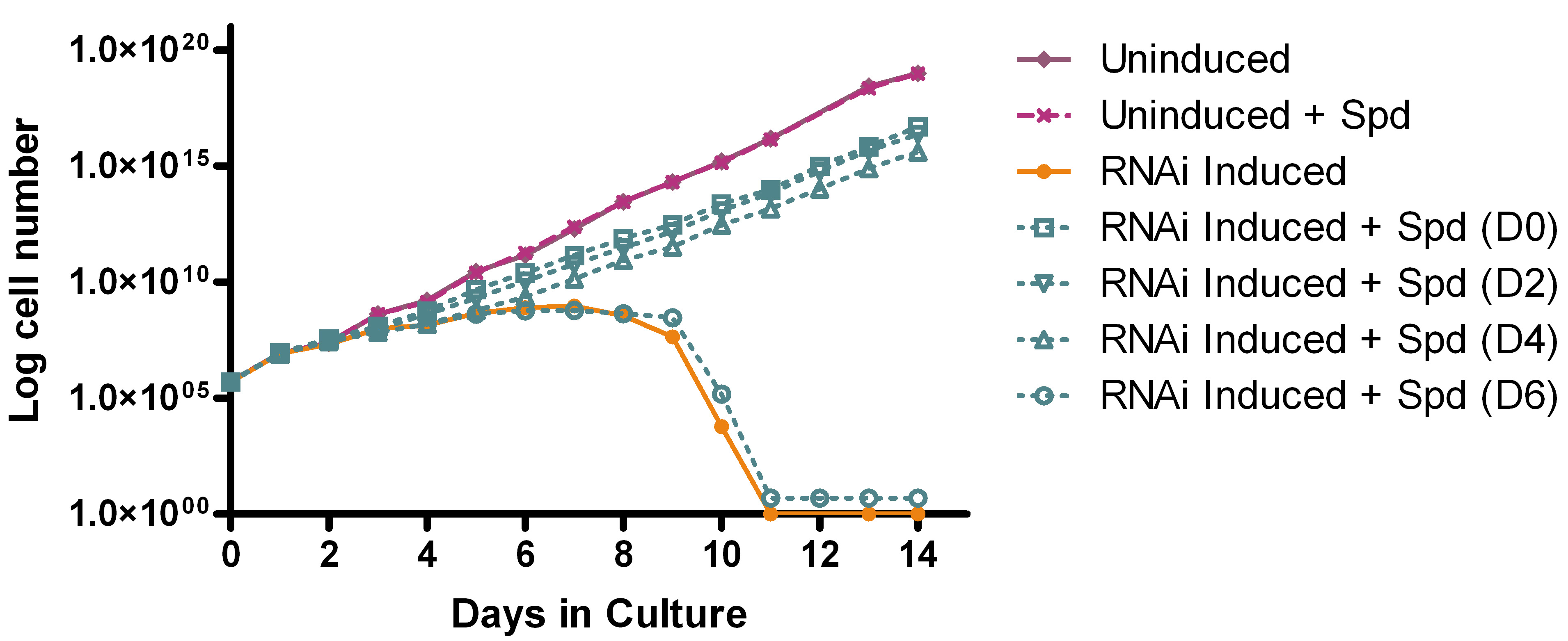

Supplement: Figure S2 — AdoMetDC RNAi cells can be rescued up to four days after RNAi induction. Spermidine was added to AdoMetDC RNAi cultures at the time of Tet addition, or two four or six days post induction, and growth of the cells was monitored. (17.94 MB TIF) [file ppat.1000183.s002.tif]

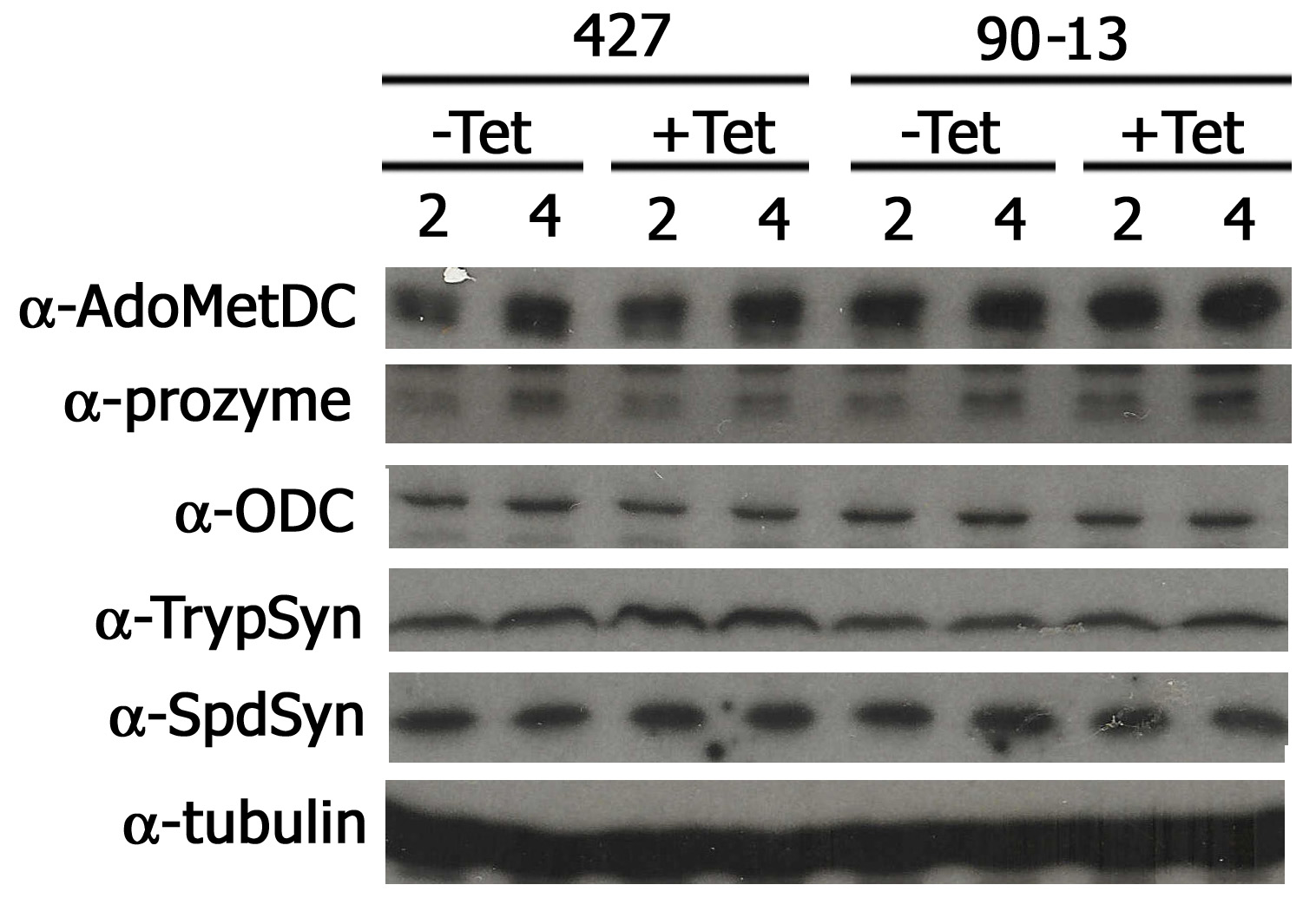

Supplement: Figure S3 — Tet treatment of blood form T. brucei control cells does not lead to prozyme or ODC induction. Untransfected 90-13 and 427 bloodstream form cells were cultured in the presence and absence of Tet for four days. (4.73 MB TIF) [file ppat.1000183.s003.tif]

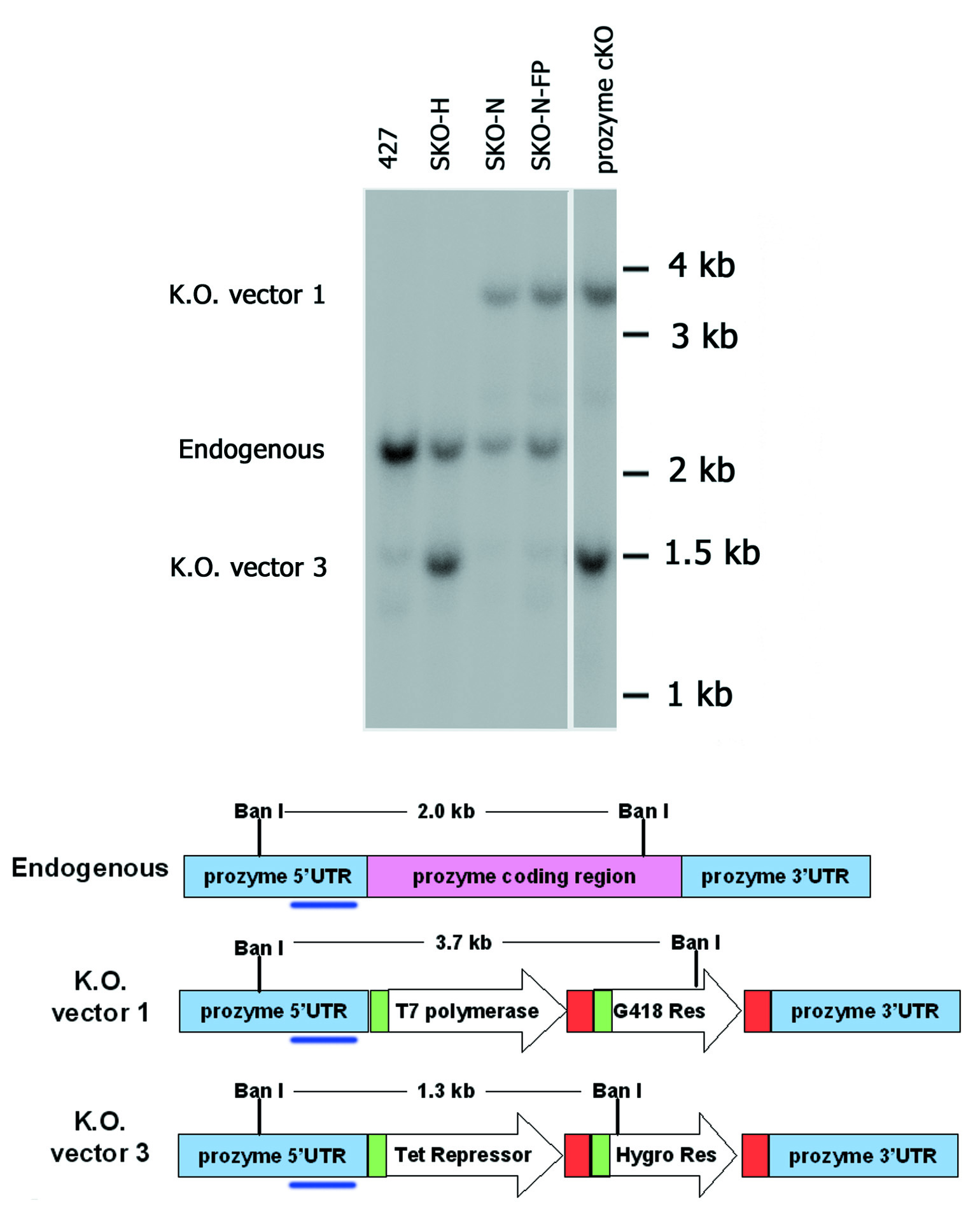

Supplement: Figure S4 — Southern blot confirmation of prozyme cKO. Genomic DNA was harvested from parental 427, single knockout SKO-N (with integrated K.O. vector 1), SKO-H (with integrated K.O. vector 3), the precursor SKO-N-FP (integrated K.O. vector 1 and exogenous FLAG-prozyme vector 2) and the prozyme cKO (integrated K.O. vector 1, integrated K.O vector 3 and exogenous FLAG-prozyme vector 2) cell lines. The genomic DNA was digested with Ban1 and probed with a region of the 5′UTR (indicated with the blue line) that is present in the endogenous locus and in each knockout vector (1 and 3, see Figure S1B). Locus representation is not to scale. (11.70 MB TIF) [file ppat.1000183.s004.tif]

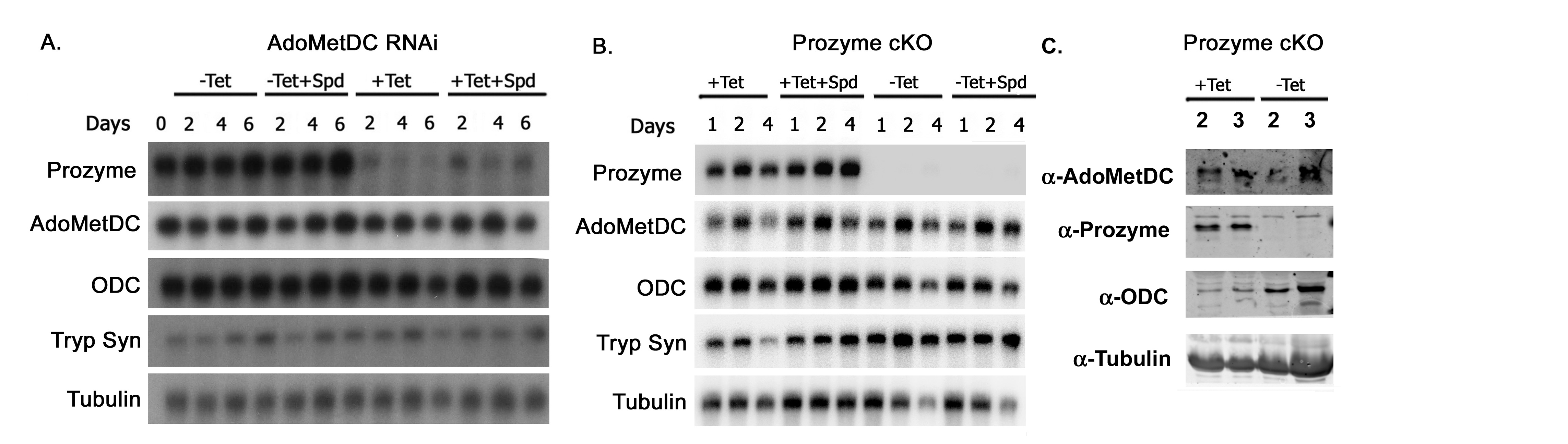

Supplement: Figure S5 — mRNA and protein levels in AdoMetDC RNAi prozyme cKO cells. Northern blot analysis of mRNA from AdoMetDC RNAi (A) or prozyme cKO (B) and Western analysis of prozyme cKO (C) cells in the conditions described in Figures 2 and 3. For Northern analysis, data for each set represent the results from a single gel that was stripped and reprobed for each gene. (5.95 MB TIF) [file ppat.1000183.s005.tif]
