# Supplementary material for: Regulated Expression of an Essential Allosteric Activator of Polyamine Biosynthesis in African Trypanosomes
Source: PLoS Pathog. 2008 Oct 24;4(10):e1000183. doi: 10.1371/journal.ppat.1000183 (PMC2562514; doi:10.1371/journal.ppat.1000183)
Supplement: Table S1 — Metabolite Analysis of AdoMetDC RNAi cells. (0.04 MB DOC) [file ppat.1000183.s006.doc]

|  | **nmol/10^8^ cells (% of control)** | | | |
| --- | --- | --- | --- | --- |
|  |  | **Day 2** | **Day 4** | **Day 6** |
|  | **Control** | **RNAi Induced** | | |
| **Putrescine** | 3.7 ± 0.42 | 24 ± 2.9 (650) | 28 ± 1.8 (760) | 24 ± 0.10 (650) |
| **Spermidine** | 70 ± 5.7 | 44 ± 4.8 (64) | 33 ± 3.5 (47) | 26 ± 5.2 (37) |
| **Glutathione** | 2.0 ± 0.20 | 1.5 ± 0.10 (75) | 1.6 ± 0.013 (78) | 1.5 ± 0.04 (75) |
| **GSH-Spd** | 0.33 ± 0.020 | 0.14 ± 0.001 (43) | 0.10 ± 0.01 (31) | 0.012 ± 0.004 (4) |
| **Trypanothione** | 0.37 ± 0.030 | 0.22 ± 0.060 (58) | 0.17 ± 0.01 (46) | 0.021 ± 0.004 (6) |
|  | **Control + Spd** | **RNAi induced + Spd** | | |
| **Putrescine** | 3.2 ± 0.43 (88) | 22 ± 1.6 (690) | 29 ± 0.65 (910) | 29 ± 1.1 (910) |
| **Spermidine** | 59 ± 3.5 (84) | 46 ± 0.74 (66) | 40 ± 7.5 (57) | 42 ± 8.0 (60) |
| **Glutathione** | 2.0 ± 0.12 (96) | 1.8 ± 0.11 (89) | 1.60 ± 0.15 (80) | 1.6 ± 0.070 (81) |
| **GSH-Spd** | 0.25 ± 0.020 (76) | 0.26 ± 0.040 (78) | 0.20 ± 0.070 (60) | 0.20 ± 0.020 (61) |
| **Trypanothione** | 0.29 ± 0.01 (78) | 0.25 ± 0.040 (67) | 0.29 ± 0.070 (77) | 0.25 ± 0.020 (66) |
| Control has no tet and AdoMetDC RNAi is not induced. All data were collected in biological triplicate. | | | | |
