# Supplementary material for: Regulated Expression of an Essential Allosteric Activator of Polyamine Biosynthesis in African Trypanosomes
Source: PLoS Pathog. 2008 Oct 24;4(10):e1000183. doi: 10.1371/journal.ppat.1000183 (PMC2562514; doi:10.1371/journal.ppat.1000183)
Supplement: Table S2 — Metabolite Analysis of prozyme cKO cells. (0.04 MB DOC) [file ppat.1000183.s007.doc]

|  | **nmol/10^8^ cells (% of control)** | | | |
| --- | --- | --- | --- | --- |
|  |  | **Day 2** | **Day 3** | **Day 4** |
|  | **Control** | **Not Expressed** | | |
| **Putrescine** | 1.6 ± 0.23 | 24 ± 0.93 (1500) | 16.0 ± 1.7 (1000) | 16 ± 1.3 (1000) |
| **Spermidine** | 32 ± 0.93 | 1.5 ± 0.63 (5) | 2.0 ± 0.32 (6) | 2.3 ± 0.41 (7) |
| **Glutathione** | 2.3 ± 0.55 | 0.68 ± 0.05 (30) | 1.2 ± 0.08 (53) | 0.85 ± 0.06 (38) |
| **GSH-Spd** | 0.23 ± 0.040 | 0.0060 ± 0.0050 (2) | 0.020 ± 0.020 (8) | 0.0020 ± 0.0010 (1) |
| **Trypanothione** | 0.18 ± 0.020 | 0.014 ± 0.0020 (8) | 0.020 ± 0.010 (11) | 0.0060 ± 0.0010 (3) |
|  | **Control + Spd** | **Not Expressed + Spd** | | |
| **Putrescine** | 1.9 ± 0.10 (120) | 12 ± 0.80 (750) | 18 ± 1.34 (1100) | 12 ± 1.0 (750) |
| **Spermidine** | 32 ± 5.0 (100) | 5.7 ± 0.63 (18) | 7.1 ± 1.0 (22) | 8.3 ± 1.0 (26) |
| **Glutathione** | 2.5 ± 0.050 (110) | 1.0 ± 0.09 (46) | 1.5 ± 0.16 (66) | 1.2 ± 0.06 (55) |
| **GSH-Spd** | 0.29 ± 0.030 (130) | 0.0040 ± 0.0020 (2) | 0.010 ± 0.0020 (7) | 0.010 ± 0.0010 (6) |
| **Trypanothione** | 0.20 ± 0.010 (110) | 0.0090 ± 0.0020 (5) | 0.010 ± 0.0030 (6) | 0.010 ± 0.0020 (4) |
| Control has tet and the regulated prozyme is expressed. All data were collected in biological triplicate. | | | | |
