# Supplementary material for: Regulated Expression of an Essential Allosteric Activator of Polyamine Biosynthesis in African Trypanosomes
Source: PLoS Pathog. 2008 Oct 24;4(10):e1000183. doi: 10.1371/journal.ppat.1000183 (PMC2562514; doi:10.1371/journal.ppat.1000183)
Supplement: Table S3 — Quantification of protein and mRNA levels. (A) Normalized ratio of protein to tubulin. AdoMetDC RNAi and 90-13 parental cells were normalized to the uninduced controls grown in the same condition. MDL 73811 treated cells were normalized to the average of untreated 427 controls collected at the identical time point (24 hours). (B) Normalized ratio of mRNA to tubulin. Data were analyzed as in (A). (C) Normalized ratios of protein to mRNA. Ratios of the values for protein/tubulin to mRNA/tubulin are displayed. (0.06 MB DOC) [file ppat.1000183.s008.doc]

A.

|  | **AdoMetDC** | **prozyme** | **ODC** | **TrypSyn** | **SpdSyn** | **TrypRed** |
| --- | --- | --- | --- | --- | --- | --- |
| **AdoMetDC RNAi** | | | | | | |
| **Uninduced** | 1.0 | 1.0 | 1.0 | 1.0 | 1.0 | 1.0 |
| **Induced D2** | 0.3 | 5.7 | 2.0 | 1.1 | 0.9 | 1.0 |
| **Induced D4** | 0.03 | 14.0 | 13.0 | 1.3 | 1.1 | 1 |
| **Induced D6** | 0.2 | 7.6 | 12.0 | 2.1 | 1.7 | 1.3 |
| **Uninduced + Spd** | 1.0 | 1.0 | 1.0 | 1.0 | 1.0 | 1.0 |
| **Induced +Spd D2** | 0.3 | 6.1 | 2.0 | 1.5 | 1.5 | 1.7 |
| **Induced +Spd D4** | 0.3 | 10.0 | 8.1 | 1.5 | 1.9 | 1.9 |
| **Induced +Spd D6** | 0.4 | 8.5 | 7.6 | 1.2 | 1.4 | 1.5 |
| **90-13** | 1.1 | 0.8 | 0.8 | 0.9 | 0.9 | 1.0 |
| **427** | 1.0 | 1.0 | 1.0 | 1.0 | 1.0 | 1.0 |
| **427 + 25 nM MDL** | 0.6 | 9.8 | 5.5 | 0.8 | 1.6 | 1.2 |
| **427 + 75 nM MDL** | 0.5 | 7.4 | 5.8 | 1.0 | 2.1 | 0.7 |

**B.**

|  | **AdoMetDC** | **prozyme** | **ODC** |
| --- | --- | --- | --- |
| **AdoMetDC RNAi** | | | |
| **Uninduced** | 1.0 | 1.0 | 1.0 |
| **Induced D2** | 0.3 | 1.2 | 1.1 |
| **Induced D4** | 0.2 | 1.7 | 0.8 |
| **Induced D6** | 0.03 | 1.2 | 0.7 |
| **Uninduced + Spd** | 1.0 | 1.0 | 1.0 |
| **Induced +Spd D2** | 0.3 | 1.2 | 1.0 |
| **Induced +Spd D4** | 0.2 | 1.4 | 0.8 |
| **Induced +Spd D6** | 0.2 | 1.2 | 0.7 |
| **90-13** | 1.0 | 0.9 | 1.0 |
| **427** | 1.0 | 1.0 | 1.0 |
| **427 + 25 nM MDL** | 1.3 | 1.6 | 1.1 |
| **427 + 75 nM MDL** | 1.5 | 2.0 | 1.3 |

**C**.

|  | **AdoMetDC** | **prozyme** | **ODC** |
| --- | --- | --- | --- |
| **AdoMetDC RNAi** | | | |
| **Uninduced** | 1.0 | 1.0 | 1.0 |
| **Induced D2** | 0.9 | 4.3 | 1.3 |
| **Induced D4** | 0.2 | 7.3 | 11.0 |
| **Induced D6** | 4.8 | 6.1 | 13.0 |
| **Uninduced + Spd** | 1.0 | 1.0 | 1.0 |
| **Induced +Spd D2** | 1.1 | 5.2 | 2.0 |
| **Induced +Spd D4** | 1.4 | 7.1 | 10.0 |
| **Induced +Spd D6** | 1.5 | 7.0 | 10.0 |
| **90-13** | 1.2 | 0.9 | 0.8 |
| **427** | 1.0 | 1.0 | 1.0 |
| **427 + 25 nM MDL** | 0.5 | 7.8 | 5.8 |
| **427 + 75 nM MDL** | 0.3 | 4.8 | 5.2 |
