# Supplementary material for: Regulated Expression of an Essential Allosteric Activator of Polyamine Biosynthesis in African Trypanosomes
Source: PLoS Pathog. 2008 Oct 24;4(10):e1000183. doi: 10.1371/journal.ppat.1000183 (PMC2562514; doi:10.1371/journal.ppat.1000183)
Supplement: Table S4 — Protein Induction: Fold change over controls. AdoMetDC RNAi (n = 3) and Prozyme cKO samples (n = 2) were analyzed by quantitative Western as described in the manuscript. Reported error represents the standard error of the mean. Tet was added (AdoMetDC RNAi) or removed (prozyme cKO) on day (D) zero, samples were collected on D2–D6. (0.03 MB DOC) [file ppat.1000183.s009.doc]

|  | **Prozyme** | | **ODC** | |
| --- | --- | --- | --- | --- |
|  | **Range** | **Average** | **Range** | **Average** |
| **AdoMetDC RNAi** | | | | |
| **Induced D2** | 6 - 19 | 14 ± 4 | 1 - 2 | 2 ± 0.4 |
| **Induced D4** | 14 - 39 | 25 ± 8 | 5 - 13 | 8 ± 3 |
| **Induced D6** | 8 - 23 | 15 ± 4 | 4 - 12 | 7 ± 2 |
| **Induced +Spd D2** | 6 - 11 | 9 ± 1 | 1 - 2 | 2 ± 0.2 |
| **Induced +Spd D4** | 8 – 11 | 10 ± 1 | 2 - 8 | 4 ± 2 |
| **Induced +Spd D6** | 6 – 20 | 11 ± 4 | 3 - 8 | 5 ± 1 |
| **Prozyme cKO** | | | | |
| **Not Expressed D2** |  |  | 3 - 5 | 4 ± 0.9 |
| **Not Expressed D3** |  |  | 3 - 7 | 5 ± 2 |
