# Supplementary material for: Regulated Expression of an Essential Allosteric Activator of Polyamine Biosynthesis in African Trypanosomes
Source: PLoS Pathog. 2008 Oct 24;4(10):e1000183. doi: 10.1371/journal.ppat.1000183 (PMC2562514; doi:10.1371/journal.ppat.1000183)
Supplement: Table S5 — Oligonucleotide primers. (0.03 MB DOC) [file ppat.1000183.s010.doc]

| **Gene/locus** | **vector** | **sequence (5' to 3')** |
| --- | --- | --- |
| **AdoMetDC RNAi construct** | | |
| AdoMetDC RNAi Forward-A | pJM 326 | ACGAGCGAAGCTTCGAAGGGCCTGAGAAGC |
| AdoMetDC RNAi Reverse-A | pJM 326 | GAAGGATCCATGAATCAGCTAGCAACTTCACTGAGC |
| AdoMetDC RNAi Forward-B | pLew100 | AAAGCACGAGCGACGCGTCGAAGGGCCTGAGAAGC |
| AdoMetDC RNAi Reverse-B | pLew100 | GAAGGATCCATGAATCATCTAGAACTTCACTGAGC |
| **prozyme cKO constructs** | | |
| prozyme 5'UTR forward | pLEW 13 and pLew 90 | ATAGCGGCCGCGATGAGCGCCAATAAGAGTTTTACC |
| prozyme 5'UTR reverse | pLEW 13 and pLew 90 | ATAACGCGT*CTCGAG*TCCCTCTGGTGTAAAACCAG |
| prozyme 3'UTR forward | pLEW 13 and pLew 90 | ATATCTAG*ATTTAAAT*GGTCACCCTGGTCAGCGCG |
| prozyme 3'UTR reverse | pLEW 13 and pLew 90 | ATAAGGCCT*GCGGCCGC*GGAGCAACTCAATAAAC |
| FLAG-prozyme forward | pLEW 300 | CCCAAGCTTATGGACTACAAAGACGATGACG |
| FLAG-prozyme reverse | pLEW 300 | CGCGGATCCTCGGCACTGCGTGCGTATGTGG |
| **Southern probe of prozyme genomic locus** | | |
| 891 bp 5' Forward |  | GCTTGGCTTCATTGATGTGCAGAAGGAGGTAGC |
| 291 bp 5' Reverse |  | CGGGTAAAACTCTTATTGGCGCTCATCGCAGG |
